# Protocol for the systematic review and meta-analysis of factors associated with non-adherence to antidepressants in depressive disorders in those more than 18 years of age

**DOI:** 10.1371/journal.pone.0281366

**Published:** 2023-02-06

**Authors:** Saraswati Dhungana, Rojal Rijal, Binit Regmi, Jala Shree Bajracharya, Subriddhi Sharma, Sunil Singh, Nilam Karn, Manisha Chapagai

**Affiliations:** 1 Department of Psychiatry and Mental Health, Institute of Medicine, Tribhuvan University, Kathmandu, Nepal; 2 Chitwan Medical College, Bharatpur, Nepal; 3 Nepalese Army Institute of Health Sciences, Kathmandu, Nepal; 4 Weifang Medical University, Weifang, China; 5 College of Medical Sciences, Bharatpur, Nepal; 6 Koshi Hospital, Biratnagar, Nepal; Xiamen University - Malaysia Campus: Xiamen University - Malaysia, MALAYSIA

## Abstract

**Objective:**

We have developed a protocol for the systematic review and meta-analysis of the factors associated with nonadherence to antidepressants in depressive patients more than 18 years of age.

**Methods:**

We will search articles with the databases PubMed- Medline, Scopus, Embase and PsychINFO. All the published literature reporting factors associated with nonadherence to antidepressants in depressive disorders in patients more than 18 years of age and fulfilling all the eligibility criteria till October 2022 will be included. The data will then be extracted and examined to be included in the systematic review. Finally, we will conduct meta-analysis for factors associated with non-adherence.

**Conclusion:**

We will do extensive search on the proposed topic within the available literature and come up with a robust review of factors associated with nonadherence to antidepressants in patients age more than 18 years globally. The evidence generated would assist in designing interventions to address non-adherence in this group of patients leading to better productivity and quality of life.

## Introduction

Depression is amongst the top leading causes of disability globally, estimated to be one of the three leading causes, alongside HIV-AIDS and Ischemic heart disease by 2030 [1, 2]. It causes huge suffering in terms of economy and productivity [1, 2]. It is a chronic condition with high relapse and recurrent rates and increased hospitalization, leading to reduced functioning and poor quality of life and increased healthcare costs [1–4]. Besides clinical and productivity issues, depression is associated with severe direct and indirect costs [1, 4–6]. Renewed evidence from the latest global burden of disease 2017 study calls for immediate action to solve the crisis since this is not just a mental health agenda but a public health problem [1]. Antidepressants are the mainstay of treatment for depressive disorders. Many patients with depressive disorders do not achieve full remission despite taking prescription antidepressants and almost half of these patients have recurrent episodes over time [7]. This requires strict adherence to treatment for patients to be able to function optimally and lead near normal lives [7].

In the last decade, there has been development of a number of safe and effective medications for depressive disorders [8]. However, studies suggest that nonadherence to antidepressants is a big challenge with estimates ranging between 13–55.7% [9] to as high as 50 to 60% [10]. Non- adherence to antidepressants further exacerbates this problem, with resultant increased relapse rates, hospitalization and poor quality of life [2]. Literature suggests a number of reasons for non-adherence, including adverse effects of drugs, nil or minimal response and other patient or doctor related factors [8, 11].

Despite being a huge problem, only scant comprehensive literature is available on non- adherence to antidepressants and factors related. A review and meta-analysis on non-adherence to psychotropics concluded that patients’ attitude towards their drugs, insight level, attitude to health and stigma mainly contributed towards nonadherence. Additionally, substance use behavior, sociodemographic variables such as age, gender, education and employment, social factors such as social support and family dynamics, clinical factors such as comorbidities and polypharmacy played roles in non-adherence to psychotropics [10]. We believe that nonadherence to antidepressants is also multifactorial [12] and similar factors account for it as supported by a recent systematic review by Solmi et al. [2, 13]. These factors have been categorized as patient- related and prescriber related [13]. A review by Chong and colleagues on interventions aimed at improving adherence to antidepressants reported that multifaceted interventions involving both service user and provider with behavioral component were effective [1], which was supported by another targeted review [13]. However, a meta-analysis published recently concluded that the interventions are effective only in the first six months [14].

To our knowledge, the only comprehensive literature on factors related to nonadherence to antidepressants in depressive disorders in form of review was published in 2015 [12] and has not been updated to this date. We, therefore, want to conduct a systematic review and meta-analysis to examine the factors associated with non-adherence to antidepressants in those more than 18 years of age.

### Research questions

What are the factors associated with nonadherence to antidepressants in depressive disorders in those more than 18 years?

### Objectives

To identify the factors associated with nonadherence to antidepressants in depressive disorders in those more than 18 years of age and conduct a meta-analysis.

## Materials and methods

### Registration and reporting of the review findings

The systematic review protocol has been registered with the PROSPERO with registration number CRD42021273639. Preferred Reporting Items for Systematic Review and Meta-Analyses: The PRISMA Statement [15, 16] and (PRISMA-P 2015) [17] will be used to report the findings of the review (S1 File).

### Study design

Systematic review and meta-analysis will be used to identify the factors associated with nonadherence to antidepressants in those with depressive disorders in patients more than 18 years of age.

### Eligibility criteria

#### Inclusion criteria

1. Studies published till October 2022.
2. All quantitative studies from cross- sectional studies to cohort studies to interventional studies and randomized controlled trials.
3. Patients diagnosed as depressive disorder/ clinical depression/ depression/ unipolar depression.
4. Studies that have looked into factors accounting for non-adherence to antidepressants.
5. Those more than 18 years of age.

#### Exclusion criteria

1. Depressive disorders not under antidepressants.
2. Depressive disorders under psychotherapy of any kind.
3. Bipolar depression.
4. Pediatric depression.
5. Editorials, systematic review, qualitative studies, viewpoint, dissertation, abstracts/presentations, and studies with incomplete data.
6. Studies conducted beyond the publication window.
7. Studies conducted in language other than English.

### PICO/S search criteria

#### Population

Patients more than 18 years of age and diagnosed as depressive disorder/ clinical depression/ depression/ unipolar depression by doctors/ clinicians/ therapists/ psychiatrists and prescribed antidepressants. We will not restrict the search to specific contexts. Individuals with comorbid conditions (e.g., diabetes, cancer, heart disease and substance comorbidities) will be included. The location (e.g., outpatient/inpatient, private practice) and mode of treatment (in person/digital), will also not restrict our search strategies.

#### Exposure

Those patients with depressive disorders and under antidepressants as prescribed by doctors.

#### Comparators

Any comparison groups of the included studies if available will be included in the review. The comparators could be any control groups or comparison groups of the intervention studies or observational studies (e.g., types of antidepressants, age group, single episode versus recurrent depressive disorder).

#### Outcome

Non-adherence to antidepressants.

Non-adherence to antidepressants as prescribed by the treating physicians will be the main outcome. For the operational definition of non-adherence, we will follow the definition given by World Health Organization, which states that non-adherence is “the extent to which a person’s behavior- taking medication, following a diet, and/or executing lifestyle changes does not correspond with agreed recommendations from a health care provider” [18]. Non-adherence to antidepressants has the potential to be labelled as dichotomous as subjective responses (Yes/ No), categorical (high, moderate, low, very low) or continuous (in terms of scores in use of scales). All studies using any assessment tool will be included in the study.

#### Studies (observational and interventional)

Studies conducted globally and available in the search databases within the publication window will be considered.

### Search strategies

Databases for search will be PubMed- Medline, Scopus, Embase and PsychINFO. We will use combination of keywords, related medical subject heading (MeSH) terms and other relevant search terms to screen the articles. The search terms used will be 1) “Factors” OR “determinants” OR “predictors” OR “causes” OR “risk factors”; 2) “Nonadherence” OR “non-adherence” OR “noncompliance” OR non-compliance” OR “adherence” OR “compliance”; 3) “antidepressants” OR “antidepressant drugs” OR “depression medication/s” OR “depression drugs” OR “drugs” OR “medications”; 4) “depressive disorders” OR “depression” OR “unipolar depression” OR “clinical depression”. The MeSH terms and keywords in title/abstract will be used in the 1+2+3+4 format in the PubMed- Medline search (S2 File). For other search databases, we will use relevant search terms with Boolean operators as applicable. Searches will be re-run before the final analysis.

#### Data extraction (selection and coding)

*Study selection*. Two reviewers (BR and RR) will independently screen and select studies to be included in the first stage using the search strategies by title and abstract based on the inclusion criteria. Discrepancies in the first stage will be resolved by third and fourth reviewers (SD and MC). Then, all the authors will independently review and agree on the full text of all selected studies to assess their suitability in the inclusion process. Zotero will be used to collect, organize, and manage literature and to keep record and manage duplicates.

#### Data extraction

Data will be extracted using an extracting data form designed for the purpose of this review by JS and SuS. All studies retrieved from our search strategies using our PICO/S search questions and inclusion criteria will be imported to Zotero and managed in different folders as necessary. All the factors associated with nonadherence as 1. patient- related factors, 2. social- economic factors, 3. health system factors, 4. condition- related factors and 5. therapy- related factors will be extracted. Below is the list of details to be included for each study included in the final stage after risk of bias assessment. In case of missing data, the authors will be contacted as necessary.

- Author and year/ date of publication
- Country and setting
- Sample size
- Participant ages
- Participant genders (N, %)
- Mean age (SD, range)
- Any comorbidity
- Antidepressant used (if stated)
- Study duration
- Research aims
- Research design
- Measures used to assess antidepressant non-adherence
- Factors associated with non-adherence
- Summarize results and key message

#### Risk of bias (quality) assessment

Standardized tools applicable to different studies as listed below will be used to assess risk of bias assessment and 2 reviewers (SS and NK) will take part in the process. Disagreements in the judgement regarding risk of bias assessment will be sorted by further discussion with SD and MC. Risk of bias assessment for randomized controlled trials version 2 (RoB 2) [19] will be used to assess risk of bias for randomized controlled studies, where we will use the tool to assess the risk in five specific areas, namely bias arising from randomization process, deviation from intended interventions, missing outcome data, measurement of outcome, and selection of reported results. ROBINS-I [20] will be used to assess risk of bias in the results of non-randomized studies that compare health effects of two or more interventions. NIH quality assessment tool (Study Quality Assessment Tools | NHLBI, NIH) or other suitable and standard quality assessment tools such as The Newcastle-Ottawa Scale (NOS) [21] will be used for specific designs of the observational studies as appropriate. ROBvis tool [22] will be used to generate traffic light plots. Grades of recommendation, Assessment, Development, and Evaluation (GRADE) [23] process will also be used eventually to check overall quality of the studies included and will be categorized as high, moderate, low and very low using GRADEpro. This will be done by SD and MC.

#### Data synthesis strategy and subgroup analysis

We plan to bring out a narrative synthesis of the results highlighting the main outcomes of interest. We will provide the measures used to assess non-adherence to antidepressants and also the factors associated with non-adherence by classifying them under specific headings identified as 1. patient- related factors, 2. social-economic factors, 3. health system factors, 4. condition-related factors and 5. therapy-related factors. Findings extracted from studies will be summarized in a summary table. A narrative synthesis of the results will be also provided and organized in topics, according to the specific exposures. The quality of the studies will be also discussed.

Our main outcome of interest is nonadherence to antidepressants. Non-adherence to antidepressants has the potential to be labelled as dichotomous as subjective responses (Yes/ No), or continuous (in terms of scores in use of scales).

For studies with dichotomous outcomes, we plan to report odd ratios, while for those with continuous outcomes, such as mean scores on adherence, we will report means for individual studies first, followed by reporting a combined effect estimate. If we find sufficient studies allowing meta-analysis, we will illustrate with a forest plot to provide an overall summary of effect estimates using odd ratios and standardized mean differences with confidence intervals for individual studies and overall.

We assume that the studies included will be heterogenous, therefore, we plan to apply random effect model. We will measure the extent of heterogeneity across the studies included using I^2^ (Inconsistency index) [24]. I^2^ 50%-90% will be considered to have substantial heterogeneity while 75% to 100% will be considered to have considerable heterogeneity. Variance within studies will be estimated by inverse variance weighting using residual maximum likelihood (REML) technique [25]. We consider doing analysis of subgroups if there are good number of studies to allow subgroup analysis for example, if adequate number of studies are available in terms of specific antidepressants used to see which group of antidepressants are associated with more nonadherence to antidepressants. We also plan to do subgroup analysis based on age groups like 18 to 45, 45 to 60 and 60 and above if this is applicable.

## Conclusion and limitations

Depressive disorders are one of the most disabling mental health conditions [26]. Evidence suggests that adherence to antidepressants is vital in preventing recurrences and lifelong disability [27]. Despite robust evidence of safe and effective drugs in most cases, nonadherence to antidepressants is amongst the big challenges faced in management of depressive disorders [28, 29]. The consequences are increased healthcare costs due to high rates of hospitalization and emergency visits, chronicity and residual symptoms, reduced functioning, disability and poor quality of life [2–5].

It is therefore imperative to understand the factors associated with nonadherence to antidepressants in patients with depression. Knowledge of the factors or determinants of nonadherence from a global perspective will help formulate treatment plans for these patients to improve the overall problem of nonadherence so as to decrease the disability caused by this condition leading to improved productivity and economic prosperity.

This systematic review, however, will not be without limitations. The biggest limitation would be the heterogeneity of study designs given the broad nature of the topic to be reviewed. Further, studies published in language other than English would be excluded and also, studies could be missed since we will not be using all the databases.

## Supporting information

S1 File PRISMA guide. (DOCX)

S2 File Search keywords. (DOCX)

## References

[1] Bonadiman CSC, Malta DC, de Azeredo Passos VM, Naghavi M, Melo APS. Depressive disorders in Brazil: results from the Global Burden of Disease Study 2017. Popul Health Metr. 2020;18(Suppl 1):6. doi: 10.1186/s12963-020-00204-5 32993670 PMC7526360

[2] Ho SC, Chong HY, Chaiyakunapruk N, Tangiisuran B, Jacob SA. Clinical and economic impact of non-adherence to antidepressants in major depressive disorder: A systematic review. Journal of Affective Disorders. 2016;193:1–10. doi: 10.1016/j.jad.2015.12.029 26748881

[3] Chong WW, Aslani P, Chen TF. Effectiveness of interventions to improve antidepressant medication adherence: a systematic review. Int J Clin Pract. 2011;65(9):954–75. doi: 10.1111/j.1742-1241.2011.02746.x 21849010

[4] Greenberg PE, Fournier AA, Sisitsky T, Simes M, Berman R, Koenigsberg SH, et al. The Economic Burden of Adults with Major Depressive Disorder in the United States (2010 and 2018). Pharmacoeconomics. 2021;39(6):653–65. doi: 10.1007/s40273-021-01019-4 33950419 PMC8097130

[5] Schofield D, Cunich M, Shrestha R, Tanton R, Veerman L, Kelly S, et al. Indirect costs of depression and other mental and behavioural disorders for Australia from 2015 to 2030. BJPsych Open. 2019;5(3):e40. doi: 10.1192/bjo.2019.26 31530305 PMC6520529

[6] König H, Rommel A, Thom J, Schmidt C, König HH, Brettschneider C, et al. The Excess Costs of Depression and the Influence of Sociodemographic and Socioeconomic Factors: Results from the German Health Interview and Examination Survey for Adults (DEGS). Pharmacoeconomics. 2021;39(6):667–80. doi: 10.1007/s40273-021-01000-1 33521892 PMC8166710

[7] Perestelo-Perez L, Perestelo P, Perez R, Serrano A, De Las C. Sociodemographic and clinical predictors of compliance with antidepressants for depressive disorders: systematic review of observational studies. Patient Preference and Adherence. 2013:151. doi: 10.2147/PPA.S39382 23487319 PMC3592507

[8] Delgado PL. Approaches to the enhancement of patient adherence to antidepressant medication treatment. J Clin Psychiatry. 2000;61 Suppl 2:6–9. 10714617

[9] Sansone RA, Sansone LA. Antidepressant adherence: are patients taking their medications? Innov Clin Neurosci. 2012;9(5–6):41–6. 22808448 PMC3398686

[10] Semahegn A, Torpey K, Manu A, Assefa N, Tesfaye G, Ankomah A. Psychotropic medication nonadherence and its associated factors among patients with major psychiatric disorders: a systematic review and meta-analysis. Systematic Reviews. 2020;9(1). 10.1186/s13643-020-1274-3 PMC6966860 31948489

[11] Párraga Martínez I, López-Torres Hidalgo J, del Campo del Campo JM, Villena Ferrer A, Morena Rayo S, Escobar Rabadán F, et al. Adherence to patients antidepressant treatment and the factors associated of non-compiance. Aten Primaria. 2014;46(7):357–66. 24704196 10.1016/j.aprim.2013.11.003 PMC6983598

[12] Martin Vazquez DMJ. Adherence to antidepressants: A review of the literature. Neuropsychiatry. 2016;6(5).

[13] Solmi M, Miola A, Croatto G, Pigato G, Favaro A, Fornaro M, et al. How can we improve antidepressant adherence in the management of depression? A targeted review and 10 clinical recommendations. Brazilian Journal of Psychiatry. 2021;43(2):189–202. doi: 10.1590/1516-4446-2020-0935 32491040 PMC8023158

[14] González de León B, del Pino-Sedeño T, Serrano-Pérez P, Rodríguez Álvarez C, BejaranoQuisoboni D, Trujillo-Martín MM. Effectiveness of interventions to improve medication adherence in adults with depressive disorders: a meta-analysis. BMC Psychiatry. 2022;22(1):487. doi: 10.1186/s12888-022-04120-w 35858887 PMC9301839

[15] Moher D, Liberati A, Tetzlaff J, Altman DG. Preferred Reporting Items for Systematic Reviews and Meta-Analyses: The PRISMA Statement. PLoS Medicine. 2009;6(7):e1000097. doi: 10.1371/journal.pmed.1000097 19621072 PMC2707599

[16] Page MJ, McKenzie JE, Bossuyt PM, Boutron I, Hoffmann TC, Mulrow CD, et al. The PRISMA 2020 statement: An updated guideline for reporting systematic reviews. PLOS Medicine. 2021;18(3):e1003583. doi: 10.1371/journal.pmed.1003583 33780438 PMC8007028

[17] Shamseer L, Moher D, Clarke M, Ghersi D, Liberati A, Petticrew M, et al. Preferred reporting items for systematic review and meta-analysis protocols (PRISMA-P) 2015: elaboration and explanation. BMJ 2015;349:g7647 doi: 10.1136/bmj.g7647 25555855

[18] World Health Organization. Adherence to long-term therapies: evidence for action / [edited by Eduardo Sabaté]. Geneva: World Health Organization; 2003.

[19] Sterne JAC, Savović J, Page MJ, Elbers RG, Blencowe NS, Boutron I, et al. RoB 2: a revised tool for assessing risk of bias in randomised trials. BMJ. 2019:l4898. doi: 10.1136/bmj.l4898 31462531

[20] Sterne JA, Hernán MA, Reeves BC, Savović J, Berkman ND, Viswanathan M, et al. ROBINS-I: a tool for assessing risk of bias in non-randomised studies of interventions. BMJ. 2016:i4919. doi: 10.1136/bmj.i4919 27733354 PMC5062054

[21] Lo CK-L, Mertz D, Loeb M. Newcastle-Ottawa Scale: comparing reviewers’ to authors’ assessments. BMC Medical Research Methodology. 2014;14(1):45. doi: 10.1186/1471-2288-14-45 24690082 PMC4021422

[22] McGuinness LA, Higgins JPT. Risk‐of‐bias VISualization (robvis): An R package and Shiny web app for visualizing risk‐of‐bias assessments. Research Synthesis Methods. 2021;12(1):55–61. doi: 10.1002/jrsm.1411 32336025

[23] Zhang Y, Akl EA, Schünemann HJ. Using systematic reviews in guideline development: The GRADE approach. Research Synthesis Methods. 2019;10(3):312–29. 10.1002/jrsm.1313 30006970

[24] Schulzke S. Assessing and Exploring Heterogeneity. Springer International Publishing; 2021. p. 33–41.

[25] Veroniki AA, Jackson D, Viechtbauer W, Bender R, Bowden J, Knapp G, et al. Methods to estimate the between-study variance and its uncertainty in meta-analysis. Research Synthesis Methods. 2016;7(1):55–79. doi: 10.1002/jrsm.1164 26332144 PMC4950030

[26] Zhao Y-J, Jin Y, Rao W-W, Zhang Q-E, Zhang L, Jackson T, et al. Prevalence of Major Depressive Disorder Among Adults in China: A Systematic Review and Meta-Analysis. Frontiers in Psychiatry. 2021;12(541). doi: 10.3389/fpsyt.2021.659470 34168579 PMC8219051

[27] Keller MB, Berndt ER. Depression treatment: a lifelong commitment? Psychopharmacol Bull. 2002;36 Suppl 2:133–41. 12490829

[28] Ho SC, Jacob SA, Tangiisuran B. Barriers and facilitators of adherence to antidepressants among outpatients with major depressive disorder: A qualitative study. PLOS ONE. 2017;12(6):e0179290. doi: 10.1371/journal.pone.0179290 28614368 PMC5470687

[29] Sirey JA, Banerjee S, Marino P, Bruce ML, Halkett A, Turnwald M, et al. Adherence to Depression Treatment in Primary Care. JAMA Psychiatry. 2017;74(11):1129. 28973066 10.1001/jamapsychiatry.2017.3047 PMC5710215

