# Supplementary material for: Protocol for the systematic review and meta-analysis of factors associated with non-adherence to antidepressants in depressive disorders in those more than 18 years of age
Source: PLoS One. 2023 Feb 6;18(2):e0281366. doi: 10.1371/journal.pone.0281366 (PMC9901773; doi:10.1371/journal.pone.0281366)
Supplement: S2 File — (DOCX) [file pone.0281366.s002.docx]

**S2 search keywords**

**Draft of search strategies to be used using PubMed Medline database**

| Component | Search terms | *Number of hits |
| --- | --- | --- |
| #1 | **(Factor*[Title/Abstract]) OR (Determinant*[Title/Abstract]) OR (Predictor*[Title/Abstract]) OR (Cause*[Title/Abstract]) OR (Risk factor*[Title/Abstract])** | 6131843 |
| #2 | **("Patient Compliance"[MeSH]) OR (Adherence[Title/Abstract]) OR (Compliance[Title/Abstract]) OR (Nonadherence[Title/Abstract]) OR (Non-Compliance[Title/Abstract]) OR (Non-adherence[Title/Abstract]) OR (Non-compliance[Title/Abstract])** | 290047 |
| #3 | **("Antidepressive Agents"[MeSH]) OR (Antidepressants[Title/Abstract]) OR (Antidepressant[Title/Abstract]) OR (Drug*[Title/Abstract]) OR (Medication*[Title/Abstract])** | 2112635 |
| #4 | **("Depressive Disorder"[MeSH]) OR (Depression[Title/Abstract]) OR (Unipolar depression[Title/Abstract]) OR (Clinical depression[Title/Abstract])** | 407511 |
| #5 | #1AND #2 AND #3 AND #4 | 1995 |
