# Supplementary material for: Protocol for the systematic review and meta-analysis of factors associated with non-adherence to antidepressants in depressive disorders in those more than 18 years of age
Source: PLoS One. 2023 Feb 6;18(2):e0281366. doi: 10.1371/journal.pone.0281366 (PMC9901773; doi:10.1371/journal.pone.0281366)
Supplement: Attachment — Submitted filename: Response to reviewers.docx [file pone.0281366.s004.docx]

18 January 2023

Response to reviewer

Reviewer #2: Thank you for addressing the comments and suggestions provided by the reviewers. The responses are comprehensive and adequately address the reviewer recommendations. I have some further very minor suggestions (line numbers refer to the tracked version of the manuscript)

Abstract
Line 43-44: please consider removing “and illustrate the results with a forest plot using Stata or Revman” as I do not think it’s necessary in the abstract. For your consideration

Authors’ response: Thank you for the comment. We agree with it and have removed “and illustrate the results with a forest plot using Stata or Revman” from the abstract section in lines 43-44 in tracked version.

Line 255: Will you be using odds or risk ratios? It doesn’t matter which, but I feel that it should be decided a priori to avoid any potential bias in the analysis
Line 260: Will you be using mean differences or standardized mean differences? Again at your preference (but I would think standardized more appropriate) but should be decided a priori.

Authors’ response: Thank you for this important observation. We agree it should be decided a priori to avoid potential bias in the analysis. We therefore have decided to use odd ratios and standardized mean differences for our statistical analysis purpose. Accordingly, we removed risk ratios and mean differences in lines 254 and 259 respectively in the new tracked version.
